# Adding eyebrows to CSCL; understanding the combined use of synchronous and asynchronous communication, and the role of motivation in computer-supported collaborative learning

**DOI:** 10.1007/s40037-014-0133-0

**Published:** 2014-08-01

**Authors:** Bas Giesbers

**Affiliations:** 1 Department of Technology and Operations Management, Rotterdam School of Management, Erasmus University Rotterdam, Burgemeester Oudlaan 50, T Building, Room T9-48, 3062 PA Rotterdam, the Netherlands; 2 School of Business and Economics, Maastricht University, Maastricht, the Netherlands

This dissertation addresses the combined use of asynchronous and synchronous communication in online computer-supported collaborative learning (CSCL) and the relation with students’ motivation. Previously, online communication had been asynchronous and text-based. Technological developments have recently made it possible to support online learning processes by the use of *rich synchronous* communication tools, such as Skype or videoconference, which resemble face-to-face interaction through (a combination of) audio, video, and chat. Combining both communication modes is a novel development that seems promising to improve CSCL. However, it also introduces further complexity to the CSCL process. The research question that is addressed in this thesis is how students use synchronous communication during weekly videoconferences in combination with posts to asynchronous discussions, and how this use relates to learner satisfaction, motivation, and performance.

Chapters 2 and 3 focus on a course level in which groups of students using asynchronous discussion forums only are compared with groups using discussion forums in combination with weekly videoconferences. Findings show that student satisfaction did not increase when using combined communication modes. However, student performance is found to be significantly *lower* in the combined setting.

Chapters 4 and 5 focus on an individual level. In Chapter 4, the actual use of synchronous communication tools (chat, audio, video) and its relation with motivation and performance is addressed. Findings show that dropout is reflected by non-participation in the videoconferences. Higher levels of intrinsic motivation are found to be associated with participation in more videoconferences, but not with the usage of more rich tools. Only the number of times students participate in the videoconferences is found to be positively associated with student performance (tool usage and motivation are not).

In Chapter 5 the dynamic interrelation of synchronous and asynchronous communication usage over time and its relation with individual motivation is investigated. Taking part in a videoconference is found to be positively related to the quantity and quality of contributions to the discussion forums. Only in the first week of the course is intrinsic motivation found to directly relate to higher quantity and quality of asynchronous communication. In following weeks, the effect of motivation on posting behaviour is shown to be indirect. From the second week onward, intrinsically and extrinsically motivated students show similar posting behaviour after participating in a videoconference. This is highly interesting, as participation in synchronous videoconferences potentially neutralizes between intrinsically and extrinsically motivated students’ contributions to asynchronous discussion forums. Further research is needed to determine if this relation is structural and causal.

